# A call for research to address the threat of paper mills

**DOI:** 10.1371/journal.pbio.3002931

**Published:** 2024-11-22

**Authors:** Jennifer A. Byrne, Anna Abalkina, Olufolake Akinduro-Aje, Jana Christopher, Sarah E. Eaton, Nitin Joshi, Ulf Scheffler, Nick H. Wise, Jennifer Wright

**Affiliations:** 1 NSW Health Statewide Biobank, NSW Health Pathology, Camperdown, Australia; 2 School of Medical Sciences, Faculty of Medicine and Health, University of Sydney, Camperdown, Australia; 3 Institute for East European Studies, Freie Universität Berlin, Berlin, Germany; 4 Taylor & Francis Group, Milton Park, Milton, United Kingdom; 5 FEBS, Cambridge, United Kingdom; 6 Werklund School of Education, University of Calgary, Calgary, Canada; 7 Adis Journals, Springer Nature, Auckland, New Zealand; 8 Wiley-VCH GmbH, Research Integrity & Case Resolution, Berlin, Germany; 9 Clare College, University of Cambridge, Cambridge, United Kingdom; 10 Cambridge University Press, Cambridge, United Kingdom

## Abstract

Research paper mills are covert organizations that provide low quality or fabricated manuscripts to paying clients. This Perspective, written by members of the United2Act Research Working Group, proposes five key research questions on paper mills that require resourcing and support.

## Teaser

Research paper mills are covert organizations that provide low-quality or fabricated manuscripts to paying clients. As members of the United2Act Research Working Group, we propose 5 key research questions on paper mills that require resourcing and support.

Research paper mills are unethical organizations that produce manuscripts at scale using derivative, copied, and/or fabricated text or data sets [1,2]. Manuscripts can be sold to preexisting author teams or individual authorship positions can be sold before and/or after manuscript acceptance [1–5]. Some paper mills may offer other services, including editorial handling and peer review [3,4], post-publication communications [5], and citations to their products [6].

Paper mills are likely to take deliberate steps to conceal their activities and products, while scaling both to maximize profits [1–3,5,7]. It was estimated that between 2 and 46% of manuscripts received by journals between 2019 and 2021 were produced by paper mills [2]. The analysis of a single paper mill identified 450 publications authored by more than 800 scholars from 300 universities [5]. New capabilities to fabricate research manuscripts using generative AI [8] could further scale manuscript production, while also rendering some fabricated manuscripts more challenging to detect [9].

Paper mills have been discussed in the literature since 2013 [1,10], yet their products, operations, and services remain understudied [11]. The lack of empirical research undertaken to date could partly reflect challenges in studying covert activities [12], safety risks for both participants and researchers [5], assumptions that paper mills do not affect mainstream research [11], and limited dedicated funding. However, any lack of paper mill research seems likely to underestimate the problem. For example, paper mills could target many different fields that allow papers to be produced and concealed at scale, where peer review expertise is limited [7], and replication of published results remains challenging [9]. Journal early warning lists [13] and the outputs of single paper mills [5] also encompass research fields beyond those typically linked with paper mill activity [9,11].

The lack of research on paper mills, relative to the potential size and significance of the problem, means that we need to describe knowledge gaps, necessary research and expertise, and how the resulting evidence could benefit all stakeholders who rely on publication integrity. United2Act represents an international group of stakeholders who are working together to address the challenge of paper mills. As members of the United2Act Research Working Group, we describe 5 key research questions (KQs) that we believe require resourcing and support, to improve our knowledge of paper mills and their impact on science and researchers (summarized in **Table 1**).

**Table 1:** Five key questions (KQ’s) for research to address the threat of paper mills.

Key questions	Topics	Expertise	Funding, support, enablers, outcomes
**KQ1** **What are the features of paper mill products?**	• Paper mill product features • Targeted fields, topics, methodologies • Screening tool development • Impact of GenAI	• Feature detection/sleuthing • Targeted field, topic, methodological expertise • Text mining • Screening tool development • GenAI/ LLM/ machine learning • Bibliometrics • Publishing	• Funding opportunities could be linked to bonuses for discipline-specific research, to incentivize research on paper mills • (Re)-training opportunities to scale capacity (e.g., screening tool development and application) • Public/private partnerships (e.g., screening tool application) • Scaled post-publication correction capacity required to enable research translation
**KQ2** **What is the scale of the paper mill problem?**	• Affected journals, publishers • Duration • Changes over time • Use of GenAI		
**KQ3** **How do paper mills operate and evade detection?**	• Operational models • Clients • Employees, required skills • Concealment • Collusion with brokers, researchers, journals, institutions	• Local knowledge, languages • Working with vulnerable participants • Qualitative research • Contract cheating • Global internet commerce • Criminal networks	• Minimize risks for both researchers and research participants • Consider offering incentives to research participants • Scaled post-publication correction capacity required to enable research translation
**KQ4** **To what extent are researchers and scholars aware of paper mills?**	• Researcher awareness (across research fields, settings) • Creation, translation, scaling, delivery, update of awareness and education campaigns	• Discipline expertise • Qualitative research • Student, researcher education • Communication	• Awareness and educational campaigns to be informed by evidence from KQ’s 1–3, 5 • Possibility for rapid translation through researcher training/education programmes
**KQ5** **How are paper mills affecting science and scholarship?**	• Citations of paper mill products by original publications, reviews, patents, clinical trials, research databases • Impacts on research problem selection, directions, careers, student completions	• Citation analysis • Bibliometrics • Text mining • Targeted field expertise • Qualitative research • Working with vulnerable participants	• Partnerships between citation analysis experts and researchers in targeted fields • Capacity to inform policies on delayed research career progression, student completions

## KQ1: What are the features of paper mill products?

Knowledge of the features of paper mill products enables their detection and deterrence. The largely incidental discoveries of publications from paper mills, combined with limited coordinated research across stakeholder groups, have likely produced incomplete and fragmented knowledge [9]. We need more complete and contemporary knowledge of both discipline-specific and -agnostic features of paper mill products to improve detection and deterrence.

## KQ2. What is the scale of the paper mill problem?

Once features of paper mill support are known, these features must be leveraged to inform understanding of the scale of paper mill-supported publications, across fields, topics, methodologies, and over time. Scaled temporal literature analyses can allow the discovery of new features, further informing KQ1, and answer questions such as when paper mills likely commenced using generative AI, and how their product features are likely to change [9].

## KQ3. How do paper mills operate and evade detection?

While some features of paper mill operations could be predicted by models of operational rationality [12], we currently know very little about paper mill operations. Large, well-resourced paper mills could conceivably provide more sophisticated manuscripts and services [1,5] and could represent arms of other unethical businesses [1,14]. As some information on paper mill operations is now dated [1,3,10], we need research to inform current paper mill operations in different countries and settings, and the demographics of paper mill employees and clients. We also need current information about how paper mills collude with researchers, institutions, journals, and/or publishers to scale operations and evade detection, including the possible use of generative AI and commercially available screening tools. Understanding how paper mills produce manuscripts could also valuably inform product features (KQ1) and problem scale (KQ2).

## KQ4. To what extent are researchers and scholars aware of paper mills?

While publishers are increasingly aware of paper mills [2,11], little is known about levels of awareness within other stakeholder groups. As awareness can help researchers to recognize and avoid paper mill products [7], research is required to inform the levels of awareness within different research and scholarly communities. The resulting information must then inform the design and implementation of awareness-raising campaigns and education programs, informed by evidence from KQs 1–3, and KQ5 below, to support all researchers, including those serving as journal editors and peer reviewers [2].

## KQ5. How are paper mills affecting science and scholarship?

Just as there is currently limited information about awareness, little is known about how paper mill products are impacting research and scholarship. For example, researchers could unknowingly cite paper mill products, potentially slowing research translation [9] or impacting patient care [4]. We therefore need to understand how paper mill products are cited in different fields and across different publication types and how these citations influence author, publication, and journal metrics. With improved awareness of paper mills (KQ4), researchers could also change their practices, problem choices or research directions in response to publications from paper mills, where individual responses might vary according to research field and/or career stage. At present, we have only scattered anecdotal descriptions (for example, from social media) of how some researchers have been affected or are choosing to respond.

In summary, paper mills represent a threat to genuine research and scholarship that requires expertise, resources, and time [9]. In any call for research on paper mills, it is important to recognize ongoing efforts to address this problem. For example, journals and publishers are leveraging known paper mill features to detect submissions and retract publications, where cross-publisher collaborations such as the STM Integrity Hub provide education, shared tools, and workflows [2,9,11]. At the same time, paper mill product features and operations could rapidly evolve in response to improved detection and new capabilities [2,9,11]. Publishers and researchers alike will therefore benefit from timely research on paper mill products and operations to improve both awareness and responses (**Table 1**).

We recognize that paper mills represent a challenging topic for researchers and funders alike. Nonetheless, research funders and institutions must now take courageous decisions to provide the necessary resources to transform our understanding of paper mills. Dedicated funding of paper mill research will also signal that paper mills and research fraud represent legitimate, important topics. Research support must enable rapid, ambitious research at scale, matched with systems that fast-track research translation to the literature and its many user communities (**Table 1**). For example, translation of paper mill research requires faster processes for achieving post-publication corrections at scale [9]. Knowing that papers can be quickly flagged where there is strong suspicion of mill involvement will also be a powerful motivator for researchers and funding agencies, who may likewise see little point in identifying problematic papers if these papers simply remain uncorrected.

Research in all fields relies upon the integrity of the literature. Paper mills and research misinformation undermine trust in research and should therefore be recognized as major global challenges, of similar importance to emerging pandemics or climate change. We must therefore waste no time in discovering the full extent of the paper mill problem and in taking all necessary steps to protect genuine science and scholarship.
